# An overview of the use of the fracture risk assessment tool (FRAX) in osteoporosis

**DOI:** 10.1007/s40618-023-02219-9

**Published:** 2023-10-24

**Authors:** M. Schini, H. Johansson, N. C. Harvey, M. Lorentzon, J. A. Kanis, E. V. McCloskey

**Affiliations:** 1 Department of Oncology & Metabolism, Metabolic Bone Centre, Northern General Hospital, University of Sheffield, Herries Road, Sheffield, S5 7AU UK; 2 Sahlgrenska Osteoporosis Centre, Institute of Medicine, University of Gothenburg, Gothenburg, Sweden; 3 Mary McKillop Institute for Health Research, Australian Catholic University, Melbourne, Australia; 4 MRC Lifecourse Epidemiology Centre, University of Southampton, Southampton, UK; 5 NIHR Southampton Biomedical Research Centre, University of Southampton and University Hospitals Southampton NHS Foundation Trust, Southampton, UK; 6 Centre for Metabolic Bone Diseases, University of Sheffield, Sheffield, UK

**Keywords:** FRAX, Fractures, Calculator, Osteoporosis, Fracture risk, Epidemiology

## Abstract

FRAX®, a simple-to-use fracture risk calculator, was first released in 2008 and since then has been used increasingly worldwide. By calculating the 10-year probabilities of a major osteoporotic fracture and hip fracture, it assists clinicians when deciding whether further investigation, for example a bone mineral density measurement (BMD), and/or treatment is needed to prevent future fractures. In this review, we explore the literature around osteoporosis and how FRAX has changed its management. We present the characteristics of this tool and describe the use of thresholds (diagnostic and therapeutic). We also present arguments as to why screening with FRAX should be considered. FRAX has several limitations which are described in this review. This review coincides with the release of a version, FRAXplus, which addresses some of these limitations.

## Introduction

In this review, we explore the literature around osteoporosis and how FRAX has changed its management.

### Osteoporosis: definition and diagnosis

Osteoporosis, although first used to characterise post-mortem bones with hollow spaces in 1820, was first defined from a consensus group in 1993 as ‘a systemic skeletal disease characterised by low bone mass and microarchitectural deterioration of bone tissue, with a consequent increase in bone fragility and susceptibility to fracture’. Dual-energy X-ray absorptiometry (DXA) was approved for the measurement of bone mineral density (BMD) by the Food and Drug Administration (FDA) in 1988 [1]. A few years later, osteoporosis was defined by a WHO Working Group in densitometric terms as a BMD that was 2.5 standard deviations (SD) or more below the mean value of young healthy women, i.e., a T-score <− 2.5 SD [2]. This threshold would classify 30% of all postmenopausal women as having osteoporosis [3]. Osteopenia was defined as a T-score between − 1.0 and − 2.5. The recommended reference range is the Third National Health and Nutrition Examination Survey (NHANES III) database for femoral neck in white women aged 20–29 years [4, 5]. As per its definition, osteoporosis increases the risk for fractures. Major osteoporotic fractures were defined as those at the hip, clinical spine, forearm, and proximal humerus as they account for about 80% of the fractures and the majority of the economic burden. Moreover, these fractures are strongly associated with low BMD, are predictive of future fractures, and display an age-dependent pattern [6].

**Fig. 1:**
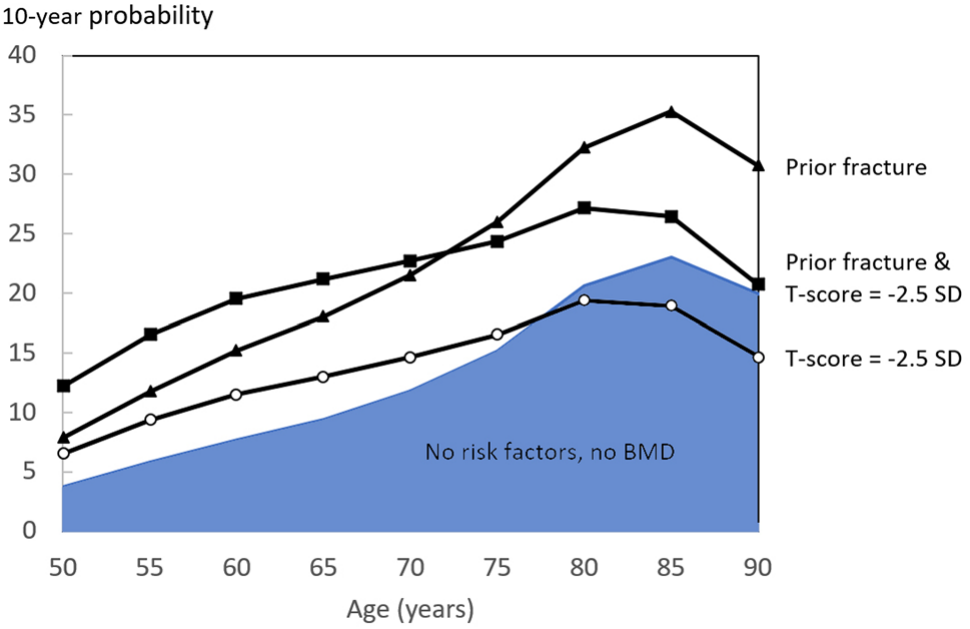
Comparison of 10-year probabilities of a major osteoporotic fracture (%) derived for a Caucasian woman in the US with a T-score of − 2.5 alone at different ages and those with a prior fracture alone, with no risk factors and unknown BMD or a combination of prior fracture and a T-score of − 2.5. From [8] by kind permission of RightsLink

The BMD-based threshold for osteoporosis, while serving a critical role in clinical diagnosis and management of osteoporosis, has several limitations which compromise its utility in identifying patients who go on to experience an incident fracture. The most important one is that although BMD has a high specificity, its sensitivity is low [2] and the majority of fractures (60–70%) occur in individuals without osteoporosis [7]. BMD might not be available or not reliable, either due to limited access to facilities or due to individual patient issues (degenerative changes at the spine, hip replacements etc.). Moreover, the significance of any given T-score threshold differs by age (Fig. 1). A T-score of − 2.5 in a woman age 65 years denotes a modest increase in the probability of fracture when compared to a woman with no clinical risk factors whose BMD is not available. With increasing age, the difference in the probability between those with T-score of − 2.5 and the general population decreases, and at older ages, for example from the age of 78 years upwards in the United States (Fig. 1), the fracture probability progressively decreases compared to the age- and sex-matched general population and a T-score of − 2.5 becomes a protective factor [8]. Furthermore, many clinical risk factors do not act solely via BMD, and thus may provide further independent contribution to risk stratification [9]. Finally, fracture rates differ between countries, and the variations cannot be explained by BMD alone. For example, the T-score corresponding to a 10-year major osteoporotic fracture probability of 20% varies from − 4.6 in Venezuela to − 2.0 in Iceland [8].

In summary, it is apparent that BMD does not capture the likelihood of fracture completely. This has led to the adverse consequence that patients at high fracture risk for non-BMD reasons have been denied appropriate treatment, because the threshold for densitometric osteoporosis has not been reached. There have been discussions whether the definition of osteoporosis should be reconsidered to include fractures [10, 11]. However, there are problems with including outcomes in multifactorial diseases. There are parallels with other multifactorial diseases like stroke. Blood pressure, a known risk factor for stroke, is a continuous variable like BMD, but not all patients with stroke have hypertension. It would be inappropriate to define hypertension on the basis of stroke. The same applies to myocardial infarction and hypercholesterolaemia [12]. A change in the BMD definition might not be widely adopted so it was recently proposed that it be retained for now and a distinction be made between diagnostic and intervention thresholds [12]. In an effort to address the insensitivity of BMD for the identification of individuals who go on to experience a fracture, risk calculators have been developed which use clinical risk factors plus/minus BMD to generate a risk of fracture over a defined period. Of these, FRAX is the most widely used, validated, and best established worldwide.

### FRAX: development and characteristics

FRAX is a computer-based algorithm that calculates the 10-year probability of a major osteoporotic fracture and a hip fracture. It was developed in 2008 by the then World Health Organization (WHO) Collaborating Centre at the University of Sheffield in the United Kingdom (UK) (http://frax.shef.ac.uk/FRAX). Through a series of extensive meta-analyses [from 12 prospective population-based studies from North America, Europe, Asia, and Australia: Rotterdam, EVOS/EPOS, CaMos, Rochester, Sheffield, Dubbo, EPIDOS, OFELY, Kuopio, Hiroshima, and two cohorts from Gothenburg, with a total of 60,000 men and women (75%) and a total follow-up of over 250,000 person years [13–18]], several risk factors for fractures were identified and incorporated into the tool. One of the main aims for the tool was for it to be easily accessible and simple-to-use in primary care. Thus, FRAX uses seven readily available dichotomous clinical risk factors (inserted as yes or no into the calculator): prior fragility fracture, parental hip fracture, smoking, systemic glucocorticoid use, excess alcohol intake, rheumatoid arthritis, and other causes of secondary osteoporosis. Other factors included in FRAX are age, sex, and body mass index (BMI). FRAX can calculate fracture probability with or without femoral neck BMD so accommodating situations where densitometric assessment is not available.

There are other well-validated assessment tools available, including the Garvan fracture calculator and QFracture. The latter (https://qfracture.org/) is a UK model [19, 20]. Similar to FRAX, it takes into account the history of smoking, alcohol, previous fracture, parental history, and glucocorticoid use. It asks specifically about several causes of secondary osteoporosis and also includes a history of falls and whether oestrogen or hormone replacement therapy (HRT) is used. BMD is not included in this tool, and it is only applicable in the UK. The Garvan tool (https://www.garvan.org.au/bone-fracture-risk) includes sex, age, weight, number of fractures since age 50 (0, 1, 2, 3, or more), and falls over the last 12 months.

A big difference of FRAX from these other tools is that FRAX calculates the probability of fracture by also considering the competing risk of mortality. This is because some risk factors (female sex, age, BMI, BMD, glucocorticoids, and smoking) affect both these outcomes. Based on that, the model integrates the hazard ratios of fracture and death [6]. The other risk engines, QFracture and Garvan, effectively report cumulative fracture incidence [21].

The impact of combining clinical risk factors and BMD can be examined using the gradient of risk, defined as the hazard ratio (HR) per SD unit change in the examined variable in the direction of increased risk (e.g., for BMD this would be a 1 SD decrease in BMD). The gradients of risk for the use of the clinical risk factors alone, femoral neck BMD alone, and the combination are shown in Table 1 [21]. Both the CRFs alone and BMD alone result in significant gradients of risk, but estimates increase when both BMD and CRFs are used, reflecting a statistically significant but relatively weak correlation between CRFs and BMD (*r* = 0.25) [21]. This correlation is, however, important to acknowledge, since it results in high FRAX probability, on average, tending to identify patients with low BMD where BMD is not included [22, 23]. FRAX has been validated using 11 independent cohorts that did not participate in the model synthesis. In all of the validation cohorts, the gradients of risk using CRFs alone or with BMD were comparable to the original ones presented in Table 1 [24].

**Table 1:** Different scenarios of gradients of risk per standard deviation change (95% confidence intervals) in risk score. Examples are given for when only bone mineral density is available, clinical factors only or the combination of the two. From [21] with kind permission from Springer Science and Business Media

Age	BMD only	Gradient of risk	
		Clinical risk factors alone	Clinical risk factors + BMD
(a) Hip fracture			
50	3.68 (2.61–5.19)	2.05 (1.58–2.65)	4.23 (3.12–5.73)
60	3.07 (2.42–3.89)	1.95 (1.63–2.33)	3.51 (2.85–4.33)
70	2.78 (2.39–3.23)	1.84 (1.65–2.05)	2.91 (2.56–3.31)
80	2.28 (2.09–2.50)	1.75 (1.62–1.90)	2.42 (2.18–2.69)
90	1.70 (1.50–1.93)	1.66 (1.47–1.87)	2.02 (1.71–2.38)
(b) Other osteoporotic fractures			
50	1.19 (1.05–1.34)	1.41 (1.28–1.56)	1.44 (1.30–1.59)
60	1.28 (1.18–1.39)	1.48 (1.39–1.58)	1.52 (1.42–1.62)
70	1.39 (1.30–1.48)	1.55 (1.48–1.62)	1.61 (1.54–1.68)
80	1.54 (1.44–1.65)	1.63 (1.54–1.72)	1.71 (1.62–1.80)
90	1.56 (1.40–1.75)	1.72 (1.58–1.88)	1.81 (1.67–1.97)

To develop a country-specific model, which is necessary because age-specific rates of fracture and death differ, data on the number of hip fractures from national sources and mortality rates from United Nation sources need to be carefully collected. Therefore, the model is as reliable as the data collected and its validation depend on the representativeness of the population. The model would ideally need rates on both hip fracture and MOF, but the latter are only available in a small number of countries, so most models use hip fractures and calculate other rates using ratios derived from Swedish data [25]. FRAX outputs are calibrated to the fracture and death rates in individual country models, so that if all the population underwent assessment by FRAX, the number of fractures predicted in the next 10 years would be equal to the observed number.

The FRAX tool was validated in the UK in a prospective cohort of 454,499 women aged 40–85 years and 424,336 men from 357 general practices and was found to be well calibrated, as the incidences of fractures predicted by FRAX were similar to those observed in the cohort. The area under the receiver-operating characteristic curve (ROC) for FRAX in hip fracture prediction was 0.85 for women and 0.82 for men [19]. FRAX without BMD was also evaluated in Norway; the study found a generally good level of agreement between the observed number of hip fractures and the predicted ones [AUC was 0.81, 95% confidence intervals (CI) 0.78–0.83 for women and 0.79 (0.76–0.83) for men] [26]. Similar numbers for hip fractures were observed in studies from Israel [27, 28] and Canada [29].

### The use of FRAX for the treatment of patients at increased risk of fractures

#### FRAX and the use of thresholds

The importance of FRAX is reflected by its inclusion in many international guidelines [30]. There are more than 150 guidelines published and FRAX is the tool used in more than half of them [31]. However, the way this tool is used for deciding whether to treat a patient or not varies among countries. In general, there are two main approaches. Thus, many guidelines use fixed probability thresholds as intervention thresholds, applied to both sexes and irrespective of age. This approach used in USA and Canada incorporates a 20% FRAX 10-year probability of a major osteoporotic fracture as the intervention threshold. Such probability thresholds vary from 4 to 20% for a major osteoporotic fracture and 1.3–5% for hip fracture, and in some cases, a 20% threshold appears to have been used principally on the basis that it is the threshold chosen in USA, rather than being appropriate for the background population fracture probability of that country [30]. The second approach is to use age-dependent thresholds, as espoused by European guidance from the International Osteoporosis Foundation and European Society for Clinical and Economic Aspects of Osteoporosis, Osteoarthritis and Musculoskeletal Diseases [32], and incorporated in other country-specific recommendations [30, 33]. Finally, there are countries that used hybrid thresholds, i.e., a combination of age-dependent thresholds and fixed thresholds. Countries following this approach include the UK National Osteoporosis Guideline Group guideline (NOGG), the Lebanon osteoporosis guideline, and the Chilean guideline [34].

The USA guidelines have affected other countries [30]. The National Osteoporosis Foundation (NOF) [now known as Bone Health and Osteoporosis Foundation (BHOF)] guidelines recommend initiation of treatment in patients with hip or vertebral fractures, patients whose T-score is in the osteoporotic range at the femoral neck, total hip or lumbar spine as assessed by DXA, and in postmenopausal women and men older than 50 years with osteopaenia and a 10-year major osteoporosis fracture probability assessed by the appropriate FRAX tool of ≥ 20% or a hip fracture probability ≥ 3% [35]. The development of these thresholds was based on an economic analysis [36]. As mentioned above, other countries have implemented this threshold without undertaking a similar analysis [30]. In other cases, for example China and Japan, thresholds have been adapted to (usually lower) mean fracture incidence in the population [37, 38].

**Fig. 2:**
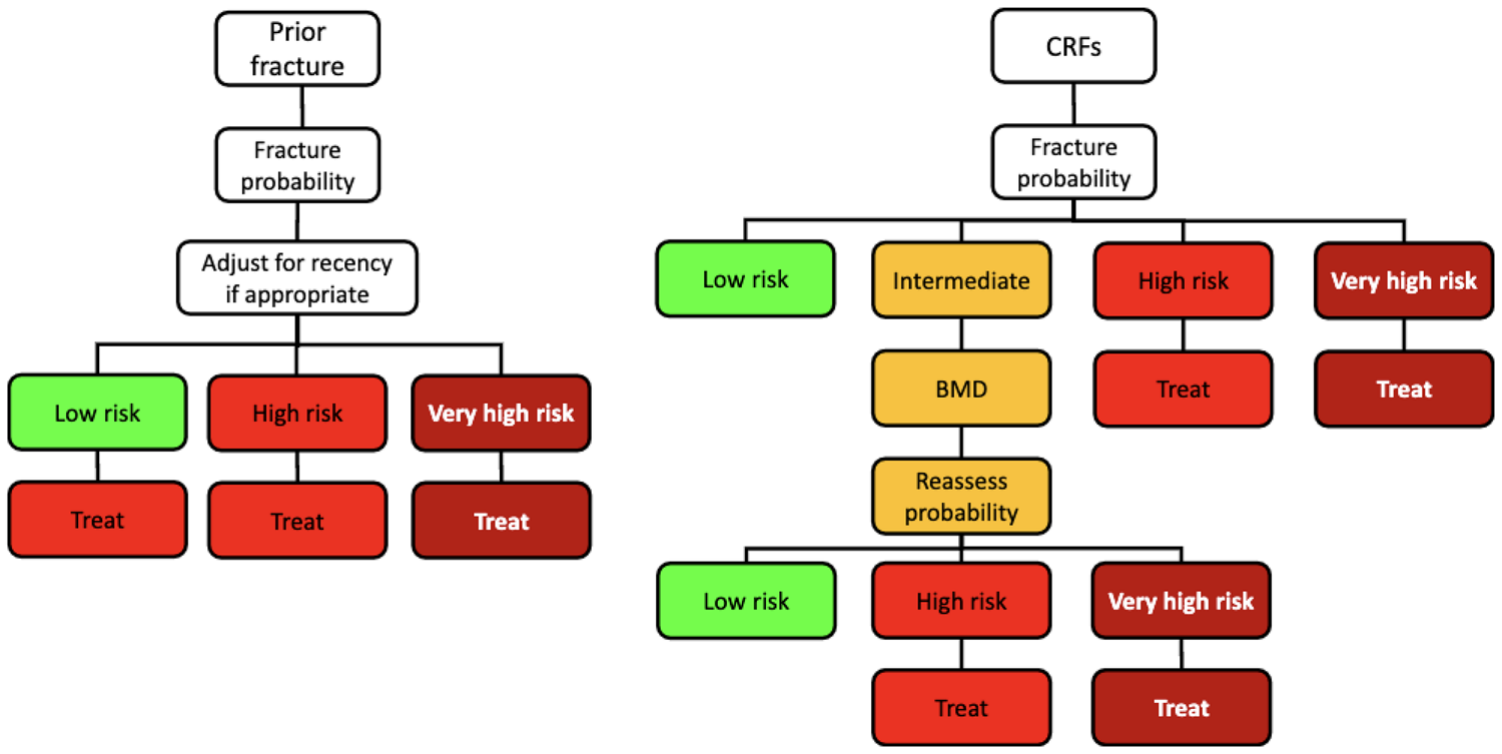
Updated algorithm for the assessment of the risk of fractures, introducing the concept of very high-risk patients. BMD bone mineral density; CRF: clinical risk factor. From [45] with kind permission from Springer Science and Business Media

Age-dependent thresholds, as espoused by the European guidance, are incorporated in recommendations from the UK National Osteoporosis Guideline Group (NOGG), representing the first national guideline to adopt this approach shortly after FRAX was introduced [39, 40]. Prior to the NOGG guidance, the Royal College of Physicians (RCP) guidance recommended the use of BMD as the basis for intervention in postmenopausal women without fracture. The notion that postmenopausal women with a prior fragility fracture should be considered for treatment without the need for a BMD measurement remains a key recommendation. Indeed, the probability of future fracture at any particular age conferred by a prior fracture, with average body mass index and no other risk factors considered, is set as the intervention threshold in the NOGG approach, as proposed through the European guidance [12]. The intervention threshold for women was also applied to men, since the effectiveness and cost-effectiveness of interventions are generally similar to that in women for equivalent risk [41]. Definition of the intervention threshold also informs the setting of assessment thresholds. The lower assessment threshold, below which BMD measurement was not needed, was based on the previous RCP and European guidelines [41, 42]. An example includes a menopausal woman, with normal BMI with no clinical risk factors. The upper threshold was chosen to minimise the probability that a patient characterised to be at high risk on the basis of CRFs alone would be reclassified to be at low risk with additional information on BMD [23]. The upper assessment threshold was set at 1.2 times the intervention threshold [41]. In the first version of this approach, a patient should first be managed by calculating their fracture probability based on age, sex, BMI, and CRFs and the fracture risk was categorised in three groups: high, low, and intermediate (Fig. 2). The high-risk group consisted of patients in whom treatment could be initiated without the need for a BMD scan, e.g., a prior fragility fracture. The intermediate group should be then assessed further by BMD to decide on the need for treatment. The RCP and original NOGG guidelines were compared, and it was found that the latter used DXA resources more efficiently. At the age of 50 years, the NOGG guidance required only 3.5 scans to identify one hip fracture, versus the RCP which required 13.9. The respective numbers at age 75 years were 0.9 and 1.5 [43].

Further developments have followed the initial implementation of age-dependent thresholds. For example, in the UK, the threshold was flattened from the age of 70 years and upwards. This was done due to the fact that the previous thresholds required a higher risk of fracture, particularly hip fracture, for treatment to be considered in older women without a prior fracture than those qualifying on the basis of fracture alone [44]. In addition, in 2020, the International Osteoporosis Foundation (IOF) and the European Society for Clinical and Economic Evaluation of Osteoporosis and Osteoarthritis (ESCEO) introduced the concept of ‘very high risk’ patients (Fig. 2). Examples of the latter include patients with recent fractures, particularly of the spine or hip, or a combination of multiple risk factors, i.e., age > 70y, prior fracture, and family history of hip fracture (Fig. 2). In these cases, osteoanabolic treatment should be considered [32, 45]

### Baseline fracture risk and treatment efficacy

One important issue to consider is whether the baseline fracture risk defines the treatment efficacy. To respond to this question, a series of post hoc analyses of clinical trials of anti-osteoporosis medications have been performed. From some medications, there was no interaction between treatment and baseline fracture risk, meaning that treatment is effective irrespective of the baseline FRAX probability. These studies included medications like abaloparatide [Abaloparatide Comparator Trial in Vertebral Endpoints (ACTIVE) study [46]], raloxifene [Multiple Outcomes of Raloxifene Evaluation (MORE) [47]], teriparatide [[48]; Teriparatide Once-Weekly Efficacy Research (TOWER) [49]], alendronate [Fracture Intervention Trial (FIT) [50]], hormone replacement treatment [51], and strontium ranelate [Spinal Osteoporosis Therapeutic Intervention (SOTI) and Treatment of Peripheral Osteoporosis (TROPOS) [52]]. Medications which showed greater efficacy with higher baseline risk included the oral bisphosphonate, clodronate [53], studied in a population sample of older women; there was a significant interaction between baseline FRAX without BMD and treatment efficacy on osteoporotic fractures, *p* = 0.043, with a weakening of this interaction when FRAX included BMD (*p* = 0.10). In this analysis, there was also a significant interaction of treatment with clodronate and BMI, which could be the major driver of the interaction with FRAX [53]. Denosumab [Fracture Reduction Evaluation of Denosumab in Osteoporosis Every 6 Months (FREEDOM)], showed no significant interaction between treatment effect and baseline FRAX probability (*p* = 0.72); however, a cubic spline function was found to give a significantly (*p* < 0.001) better fit with a greater effect at higher probabilities [54]. Bazedoxifene showed no overall interaction between treatment and FRAX with BMD (*p* > 0.30), but when the 10-year probability of major osteoporotic fracture is greater than 16% (80th percentile), the treatment was associated with a significant decrease in the risk of all clinical fractures [55]. Finally, significant interactions were observed between romosozumab and baseline FRAX for clinical fractures, osteoporotic fractures, and MOF (*p* = 0.064–0.084), but not vertebral fractures (*p* > 0.30) [FRActure study in postmenopausal women with ostEoporosis (FRAME)] [56]. These observations are of significance when thinking about the cost-effectiveness of treatment. These results have a number of important implications, principally demonstrating the validity of selecting patients for treatment on the basis of fracture probability, potentially improved cost-effectiveness, and the inappropriateness of purely BMD-based thresholds [57]. Importantly, if BMD is not available, high fracture patients as characterised by CRFs and FRAX are likely to respond to treatment. Targeting some medications for high-risk fracture patients would be cost-effective if greater efficacy is assumed from such analyses [58].

### Global usage of FRAX

The importance of FRAX in the management of osteoporosis is reflected by its global usage. FRAX is available in 86 models for 78 countries [(https://frax.shef.ac.uk/FRAX/), accessed February 2023]. Over the period ranging from 15 February 2010 to 31 December 2018 inclusive, there were around 4.3 million sessions in the USA, followed by 1.3 million in the UK and 0.3 million in Canada [59]. In February to April 2020 inclusive, the website recorded 460,495 sessions from 184 countries, with the majority (29.2%) coming from USA [60]. Its usage by country model is available on the website; however, this is an underestimation, because there are other portals available to access FRAX, including BMD equipment, smartphone applications, and some healthcare electronic record systems. The COVID-19 pandemic negatively affected access to FRAX (average reduction 58%, with two-thirds of the countries having at least 50% reduction) [60].

### The use of FRAX for population screening

Although guidelines in USA and Canada suggest routine DXA assessment in older women, in practice, this tends to be prompted by the discovery of clinical risk factors rather than a systematic approach to screening. Indeed, the European approach has recommended intervention assessment and intervention on the basis of case finding. However, recent trials of screening and associated health economic analyses have generated an increasingly convincing evidence base, on which to develop approaches to systematic screening for high fracture risk at the population level. The position paper, led by the Epidemiology and Quality of Life Working Group of the International Osteoporosis Foundation has recently considered the recommendations for the adoption of national screening programmes [61]. In the UK, screening for high fracture risk is a key recommendation of the Royal Osteoporosis Society All Party Parliamentary Group in Osteoporosis report into primary prevention (chrome-extension://efaidnbmnnnibpcajpcglclefindmkaj/https://strwebprdmedia.blob.core.windows.net/media/vxuhotlh/appg-on-osteoporosis-and-bone-health-inquiry-report-into-primary-care-2022.pdf, Accessed March 2023).

The first study to address population-based screening was undertaken in the UK. The screening for prevention of fractures in older women (SCOOP) trial was designed to evaluate whether a community screening programme based on FRAX hip fracture probability could reduce the incidence of fractures in older women (70–85 years) over a period of 5 years. This was a pragmatic, unblinded, two-arm, parallel, randomised-controlled trial which recruited women from 100 general practitioner (GP) practices from around Birmingham, Bristol, Manchester, Norwich, Sheffield, Southampton, and York. Women known to be on treatment for osteoporosis (other than calcium and vitamin D) were excluded. Other diseases or factors which could make participants unsuitable for the study were taken into account and patients were excluded on the basis of these (advanced malignancy, dementia, and recent bereavement) [62]. Women were randomised either to the screening arm (*n* = 6233) or to the usual care arm (*n* = 6250) which included opportunistic case finding. In the screening arm, FRAX probabilities were calculated and the 10-year probabilities for a hip fracture were compared with assessment thresholds [63], to determine whether a BMD measurement was needed (*n* = 3064, 49%). FRAX hip fracture probability calculated with BMD was then used to separate patients into high and low fracture risk groups. High-risk patients (*n* = 898, 14%) were invited to discuss treatment options with their general practitioner. By the end of year one, 953 subjects (15%) in the screening arm were given a prescription for an anti-osteoporosis medication. The respective number for the control arm was 264 (4%). There was a steady increase of use of medications in the control arm with time. While the study concluded that screening did not reduce the incidence of all osteoporosis-related fractures [hazard ratio (HR) 0.94, 95% confidence intervals (CI) 0.85–1.03, *p* = 0.178], nor the overall incidence of all clinical fractures (0.94, 0.86–1.03, *p* = 0.183), the approach led to a 28% relative reduction in hip fractures (prespecified secondary outcome) compared with usual care (2.6% vs 3.5%; HR 0.72, 95% CI 0.59–0.89, *p* = 0.002) [64]. This effect on hip fracture was found to increase significantly with increasing 10-year hip probability at baseline [65], consistent with the treatment effect being mediated through prescription of anti-osteoporosis medications in such women. Women in the screening group showed greater adherence with medication [66]. Screening was found to be cost-effective [67, 68].

Around the same time, the SCOOP trial, the Risk-stratified Osteoporosis Strategy Evaluation (ROSE) study followed a similar approach in women aged 65–80 years in Denmark. Participants were randomised to either screening or control. The difference with SCOOP is that DXA in the screening group, was only performed if the 10-year probability of major osteoporotic fracture was over or equal to 15% and then only those who had osteoporosis as defined by the T-score were offered treatment [69]. As a result, the study failed to show a benefit on the fracture incidence after 5 years of follow-up. However, the screening strategy was found to be beneficial in women with moderate or high risk in the per-protocol analysis (post hoc analysis). In this analysis, women in the screening group who had an FRAX score ≥ 15% and were scanned, were compared to women in the control group who had a FRAX score ≥ 15% but were not scanned. The women in the first group had fewer incident fractures and the risk reduction with a greater effect on hip fractures (adjusted sub-hazard ratio 0.741, *p* = 0.007) [70].

Finally, the SALT Osteoporosis Study (SOS) in the Netherlands assessed women aged 65–90 years having at least one CRF (unlike the SCOOP and ROSE studies which randomised everyone). One of the problems with this study is that the UK FRAX model was used. Treatment was given to women who were found to have high probabilities, but only in combination with a T-score ≤ − 2.0 or a prevalent vertebral fracture. The study failed to show an effect on fracture incidence. A post hoc analysis suggested that screening might be most effective after a recent fracture (HR = 0.65; 95% CI 0.44–0.96 for MOF and HR = 0.38; 95% CI 0.18–0.79 for hip fractures) [71].

A subsequent meta-analysis of all three studies showed that population screening could be effective in reducing major osteoporotic and hip fractures. For MOF, the HR was 0.91, 95% CI = 0.84–0.98, while for hip fractures, HR = 0.80; 95% CI = 0.71–0.91, i.e., a 9 and 20% reduction, respectively [72].

### Limitations of FRAX and available adjustments

Over the years, FRAX has been criticised for a number of issues, particularly that it is restricted in the number and granularity of risk factors. As mentioned above, FRAX was designed to be a simple tool, accessible, and easy to use in primary care. Therefore, only a yes or no answer is accommodated in most questions in the tool. This means that risk factors which are number- or dose-dependent are not fully captured. Examples include the number of prior fractures, the consumption of alcohol and the dose of glucocorticoids. Other concerns include the lack of provision for lumbar spine BMD and the absence of measurements of the material or structural properties of bone. Finally, the age of the parental fracture might be of interest.

Over time, a number of studies have proposed arithmetic adjustments to the conventional FRAX probabilities, to address some of these limitations. To address the issue of glucocorticoids, the guidance shown at Table 2 was proposed [73]. Recently, in the absence of a direct question related to falls history in FRAX and suggested potential adjustments [74], an analysis has provided probability ratios or multipliers that can be applied according to the number of falls over the last year [75, 76].

**Table 2:** Adjustments (percentage) of the FRAX according to dose of glucocorticoids. From [73] with kind permission from Springer Science and Business Media

Dose	Prednisolone equivalent (mg/day)	Age (years)						
		40	50	60	70	80	90	All ages
Hip fracture								
Low	<2.5	− 40	− 40	− 40	− 40	− 30	− 30	− 35
Medium^a^	2.5–7.5							
High	≥7.5	+25	+25	+25	+20	+10	+10	+20
Major osteoporotic fracture								
Low	<2.5	− 20	− 20	− 15	− 20	− 20	− 20	− 20
Medium^a^	2.5–7.5							
High	≥7.5	+20	+20	+15	+15	+10	+10	+15

^a^no adjustment

Probability ratios have also been provided according to the recency of fractures. For example, for a woman at age 50 years, a recent (0–2 years) vertebral fracture is associated with a 1.92-fold higher probability than for a woman of the same age who had a prior fracture at any time [77]. Multipliers have also been provided according to the number of fractures [75]. In the last scenario, a simple solution would be to lower the FRAX-based fracture probability by 5% (i.e., 0.95 × FRAX probability) in the presence of a single prior fracture and to elevate the probabilities by 10, 20, and 30% with a history of 2, 3, and 4 or more prior fractures, respectively.

When BMD at the lumbar spine (LS) is available, and there is discordance between this result and that at the femoral neck (FN), the following rule has been proposed: to increase/decrease FRAX estimate for a major fracture by one-tenth for each rounded T-score difference between LS and FN (10% per SD) [78]. A further study showed that adjustments for large LS/FN discrepancies (> 2SD) only impact to a large extent a relatively small number of people, whereas moderate (1–2 SD) discrepancies only have a small impact [79]. Trabecular bone score (TBS) and hip axis length are sometimes available, and adjustments of fracture probabilities have been provided [80, 81].

A few adjustments have been proposed in cases with type 2 diabetes mellitus, including adjustment with TBS or use of the rheumatoid arthritis (RA) input [82]. A similar approach (using the RA output) has been proposed for Parkinson’s disease [83]. Place of origin can also affect FRAX probabilities, as shown in a study in Sweden [84], where the hip fracture incidence for Swedish-born people was approximately double when compared to the one of people born outside the country. Moreover, there was an increase in the hip fracture incidence with time from immigration (0.6% per year). It was suggested that the country of birth should be used when using FRAX [84].

There are variables not incorporated into FRAX, but studies have shown that the fracture probabilities would remain unaffected if these were taken into account. In Canada, FRAX resulted in a robust prediction of fractures regardless of socioeconomic status, given that the competing risk of mortality is taken into account [85]. Moreover, FRAX with and without BMD was found to be unaffected by current or previous osteoporosis treatment [86] and body composition [87].

Although these adjustments can be undertaken by the user to modify the fracture probability output by FRAX, a further website, FRAXplus, will allow the user to incorporate such effect modifiers automatically.

### Development of the core FRAX tool

FRAX has been designed as a living tool, hence the adaptation to more countries and territory models, languages, etc. In addition to the ability to modify FRAX probability using the new FRAXplus website (https://www.fraxplus.org/), the availability of new cohorts, and longer follow-up in existing cohorts, will underpin the development of the second version of the core FRAX tool itself. This update of FRAX will be informed by analysis of 64 cohorts, including more than 2 million individuals, 69% of them being women [88]. It includes data on 41,015 hip fractures and 113,641 MOF. The expanded numbers will enable more accurate model development and consideration of other risk factors, for example falls and type II diabetes in the risk engine itself.

## Conclusion

FRAX is the globally leading tool for assessment of fracture risk, incorporated into over 100 guidelines internationally. In addition to informing treatment decisions, it can also be used to guide assessment with BMD most efficiently. The cost-effectiveness of such approaches has been demonstrated, as has the potential utility of FRAX as the core of approaches to population screening for high fracture risk. The new FRAXplus website will permit modification of FRAX probability to account for a range of additional clinical considerations and the second version of the core FRAX risk engine is under development.
